# Vaginal Microbiome and Pregnancy Complications: A Review

**DOI:** 10.3390/jcm13133875

**Published:** 2024-06-30

**Authors:** Angeliki Gerede, Konstantinos Nikolettos, Eleftherios Vavoulidis, Chrysoula Margioula-Siarkou, Stamatios Petousis, Maria Giourga, Panagiotis Fotinopoulos, Maria Salagianni, Sofoklis Stavros, Konstantinos Dinas, Nikolaos Nikolettos, Ekaterini Domali

**Affiliations:** 1 Unit of Maternal-Fetal-Medicine, Department of Obstetrics and Gynecology, Medical School, Democritus University of Thrake, GR-68100 Alexandroupolis, Greece; knikolet@med.duth.gr (K.N.); nnikolet@med.duth.gr (N.N.); 2 Second Department of Obstetrics and Gynecology, Medical School, Aristotle University of Thessaloniki, GR-54640 Thessaloniki, Greece; evavo@auth.gr (E.V.); margioulasiarkouc@gmail.com (C.M.-S.); petousisstamatios@gmail.com (S.P.); konstantinosdinas@hotmail.com (K.D.); 3 First Department of Obstetrics and Gynecology, Medical School, National and Kapodistrian University of Athens, GR-11528 Athens, Greece; giourgam@med.uoa.gr (M.G.); pfoteino@med.uoa.gr (P.F.); msalagianni@bioacademy.gr (M.S.); sfstavrou@med.uoa.gr (S.S.); kdomali@yahoo.fr (E.D.)

**Keywords:** vaginal microbiome, pregnancy complications, preterm birth, miscarriage, gestational diabetes mellitus, preeclampsia, chorioamnionitis, ectopic pregnancy, preterm premature rapture of membranes

## Abstract

**Background/Objectives:** There are indications that the microbial composition of the maternal mucosal surfaces is associated with adverse events during pregnancy. The aim of this review is to investigate the link between vaginal microbiome alterations and gestational complication risk. **Methods:** This comprehensive literature review was performed using Medline and Scopus databases. The following search algorithm was used, “Pregnancy Complications” [Mesh] AND (Vagin*), and after the literature screening, 44 studies were included in the final review. **Results:** The studies that were included investigated the association between vaginal microbial composition and preterm birth, miscarriage, preeclampsia, ectopic pregnancy, gestational diabetes mellitus, chorioamnionitis, and preterm premature rupture of membranes. In most of the studies, it was well established that increased microbial diversity is associated with these conditions. Also, the depletion of Lactobacillus species is linked to most of the gestational complications, while the increased relative abundance and especially *Lactobacillus crispatus* may exert a protective effect in favor of the pregnant woman. Several pathogenic taxa including *Gardnerella*, *Prevotella*, *Sneathia*, *Bacterial Vaginosis-Associated Bacteria-2*, *Atopobium*, and *Megasphera* seem to be correlated to higher maternal morbidity. **Conclusions:** Vaginal microbiome aberrations seem to have an association with pregnancy-related adverse events, but more high-quality homogenous studies are necessary to reliably verify this link.

## 1. Introduction

Maternal morbidity constitutes a public health issue of great importance because it not only undermines both the mother’s and infant’s well-being but also significantly increases the pregnancy-related incremental costs [1]. In fact, various studies have estimated that severe morbidity rates can complicate from 0.53% up to 6.78% of pregnancies taking place in hospitals globally [2,3,4,5,6,7,8] with an extensive meta-analysis calculating the overall pooled prevalence SMM at 2.45% [9]. Pregnancy-related complications include maternal hemorrhage, maternal sepsis and other pregnancy-related infections, hypertensive disorders (HD), stillbirth, miscarriage, ectopic pregnancy, gestational diabetes mellitus (GDM), and spontaneous preterm birth (sPTB). Among them, maternal hemorrhage is the most fatal, accounting for up to 27% of maternal mortality worldwide [10], while some of the most common complications are GDM and HD with a prevalence of up to 15% [11] and up to 25%, respectively [12].

Given that every pregnancy can be prone to unpredictable and sudden-onset complications, prompt access to appropriate care facilities is of paramount importance [13]. However, the ability to predict a woman’s predisposition to a certain complication based on demographic, genetic, and social data would be an essential step in pregnancy-related morbidity reduction [14]. Towards this direction points the analysis of either the mother’s or the newborn’s microorganism collections that inhabit the skin and multiple mucosal surfaces, named microbiota, to assess their association with several pregnancy-related complications [15]. The female gut, vagina, oral cavity, respiratory system, and uterus constitute major parts of the mucosal surfaces colonized by microorganisms [16]. For instance, recent advances in reproductive health indicate that preeclampsia is associated with an abnormal gut microbiome, while women with gestational hypertension have more periodontal pathogens in their oral cavity compared to normotensive control study participants [17,18].

The human vagina harbors microorganisms that protect the host from several urogenital diseases, such as urinary tract infections, sexually transmitted diseases, and bacterial vaginosis. This disease-preventing ability is attributed to the low vaginal pH due to lactic acid production by *Lactobacillus* sp., the production of bacteriostatic substances, and competitive exclusion [19,20,21]. Factors that can result in aberrations in the vaginal bacterial composition include obesity, hormonal changes, socioeconomic status, race, ethnicity, antibiotic administration, and urogenital infections [22,23].

During a non-complicated pregnancy, the vaginal microbiota remains stable with a predominance of *Lactobacillus species* [23]. Other bacteria that can be present in the vaginal microbiome include *Gardnerella vaginalis* (*G. vaginalis*), *Atopobium vaginae* (*At. Vaginae*), *Prevotella* sp., *Sneathia amnii* (*S. amnii*), and *Candidatus Lachnocurva vaginae* (*C. Lachnocurva vaginae*) [24]. To investigate more efficiently the highly diverse landscape of microbial taxa found in the vagina, a taxonomic classification into community state types (CST) based on the dominant bacterial species has been widely adopted. This classification includes CST-I (*Lactobacillus crispatus*), CST-II (*Lactobacillus gasseri*), CST-III (*Lactobacillus iners*), CST-V (*Lactobacillus jensenii*), and CST-IV, dominated by several anaerobic species [25].

Since oral cavity and gastrointestinal tract microbiome abnormalities have been associated with certain pregnancy complications, combined with evidence from single-arm studies where, in miscarriage cases, there is a diminished abundance of *Lactobacillus* sp. and an increased isolation ratio of anaerobes, it ignites many questions about the association between vaginal microbiota composition and pregnancy-related complications [17,18,26]. Thus, the aim of this literature review is to share reliable insights on the possible role of the vaginal microbiome in gestational complications and further elucidate their association.

## 2. Materials and Methods

A literature search was performed using the MEDLINE and Scopus databases. The following terms were used in the search text fields: (“Microbiota” [Mesh]) AND “Pregnancy Complications” [Mesh] AND (Vagin*). The search algorithm was adjusted for each database while maintaining a common overall architecture. Published observational and interventional studies investigating the association between vaginal microbiome and pregnancy complications published up to 22 April 2024 were included while reviews, letters, and commentaries were excluded.

Retrieved records underwent semi-automatic deduplication by Rayyan (version 1.4.3., Rayyan Systems, Inc., Cambridge, MA, USA) [27]. Unique records were imported into Rayyan. Two independent authors screened them for relevance based on titles and abstracts only. Disagreements were resolved through consensus or by discussion with a third author. Articles deemed as irrelevant were excluded and the full-text copies of the remaining articles were assessed for eligibility as per the PICOS criteria by two blinded reviewers. Inconsistencies were, once again, resolved by consensus or by a third reviewer. The references of the full-text copies were accessed to prevent the potential loss of eligible studies that were missed by the database search (snowball procedure). The following data items were extracted from the eligible studies: year of publication, study design, country, center and time period during which the study was conducted, number of participants, age, Body Mass Index (BMI), time point of vaginal swab collection, alpha diversity, beta diversity, dominant CST through the study arms, events of preterm birth (defined as the live delivery of one or more infants at less than 37 completed weeks of gestation), miscarriages (defined as the loss of pregnancy before 20 weeks of gestation), preeclampsia, and gestational diabetes mellitus.

## 3. Results

### 3.1. The Selection Process of Included Studies

**Figure 1:**
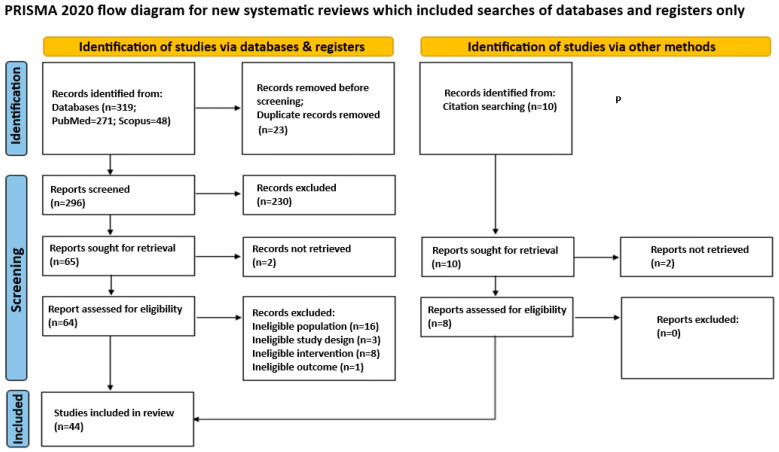
Flow diagram of the selection process of the included studies.

A flow diagram of the selection process is presented in Figure 1. In total, 319 papers were initially identified, and after duplicate removal, 296 were considered eligible for title-abstract screening. Subsequently, 66 articles were selected for full-text screening; 20 of them were excluded for the reasons presented in Figure 1, while 36 met the inclusion criteria and were included in this review. Furthermore, the references of the included studies and references from other relevant studies from high-impact journals were hand-searched and eight papers that were lost from the initial literature search were included as well. Thus, the 44 studies that were included in total investigated the association between vaginal microbial composition and preterm birth (PTB), miscarriage, preeclampsia (PE), ectopic pregnancy, gestational diabetes mellitus (GDM), chorioamnionitis (CAT), and preterm premature rupture of membranes (PPROM). A summary of the included study characteristics along with some of their most important results are provided in Table 1.

#### 3.1.1. Preterm Birth

Preterm birth is one of the leading causes of neonatal morbidity and mortality, accounting for virtually 15 million births annually worldwide [71]. Many conditions can provoke PTB, including preterm premature rapture of membranes or infections [72]. *Lactobacilli*, which are usually the predominant species in the vaginal microbiome, antagonize against dysbiosis-causing microorganisms and restrain the proliferation of anaerobes commonly correlated with bacterial vaginosis [73]. Thus, theoretically, disruption in the vaginal microbial composition could undermine the protective mechanisms against vaginal dysbiosis and consequently against PTB.

Multiple studies have inquired into the probable association between vaginal microbial composition and PTB. The largest case–control study with 449 participants (94 in the PTB arm and 355 in the healthy control arm) suggested that *Lactobacillus gasseri* (*L. gasseri*)*/Lactobacillus johnsonii* (*L. johnsonii*), *Lactobacillus crispatus* (*L. crispatus*), *Lactobacillus acidophilus* (*L. acidophilus*), *Lactobacillus iners* (*L. iners*), *Ralstonia solanacearum* (*R. solanacearum*), and *Bifidobacterium longum* (*B. longum*)*/Bifidobacterium breve* (*B. breve*) might be associated with a decreased risk of early but not late PTB. Furthermore, no correlation was found between CST assignment and PTB risk [60]. No correlation was found between specific CSTs and PTB in a retrospective study, where *Lactobacillus*-dominated communities had an inverse association with PTB in women swabbed before 12 weeks and a direct association with women swabbed at or after 12 weeks [29]. Similar results arise from a small retrospective study, a prospective study, and two case–control studies where the abundance or depletion of *Lactobacillus* in the vaginal microbiome and the CST assignment were not significantly associated with PTB [41,43,50]. Additionally, results from another case–control study indicate that, although in the term group, the richness and diversity of the microbiome remained stable throughout the course of pregnancy, while in the PTB group, both richness and diversity significantly decreased until labor, after taxonomic composition analysis, no association between any of the detected taxa and PTB reached statistical significance [58].

Given that the first indicator of important differences in microbial composition is the elevated alpha diversity, many studies have employed it as an initial step of their analysis. Starting from the alpha diversity analysis, which revealed statistically significant differences regarding richness and diversity between the groups (*p* < 0.01), higher bacterial loads and higher rates of mollicutes, meaning *Mycoplasma* and *Ureaplasma*, were spotted in the PTB compared to the control group (*p* = 0.049 and *p* = 0.012 respectively) [39]. In a prospective study, where the alpha diversity assessed through Shannon indices and the number of observed ASVs were higher in the PTB group (*p* = 0.0009 and 0.0003 respectively), increased rates of *Atopobium*, *Gardnerella*, and *Prevotella* were observed in PTB cases. In addition, after hierarchical clustering analysis, it was concluded that the PTB group was significantly more frequently assigned to CST-IV (*p* = 0.004), while at the taxa level, an increased relative abundance of *Lactobacillus* was positively associated with term birth (*p* = 0.007) [51]. Comparable outcomes emerge from a prospective cohort study where vaginal microbiotas of the healthy, uncomplicated cohort were characterized by low diversity and *Lactobacillus* dominance, while women who experienced PTB had intermediate or low profiles of *Lactobacillus* and high diversity (*p* = 0.0011), even after the analysis was adjusted for potential confounders, such as ethnicity, BMI, smoking, and medical interventions [30]. Except for *Lactobacillus* spp. depletion, increased abundance of *Gardnerella* is associated with PTB (*p* = 0.0070). In fact, using an arbitrary threshold of 70% *Lactobacillus* spp. abundance and 0.1% of *Gardnerella* abundance, the researchers calculated an OR for PTB of 5.81 (95% CI 1.12–33.7) and 5.12 (95% CI 1.05–31.1), respectively [32].

Besides the diversity and richness of the vaginal microbiome, differences seem to be present in CST assignments as well [33]. More specifically, in a prospective study, women delivering at term were mostly assigned to CST-IV, while women from the PTB arm were mostly assigned to CST-III [57]. Contradicting results emerge from a cross-sectional study where both CST-III (*L. iners*-dominated) and CST-IV were associated with spontaneous PTB with an OR as high as 4.1 (95% CI 1.1-infinity) and 7.7 (95% CI 2.2-infinity), respectively. In addition, in marginal analysis, the relative abundance of Gardnerella vaginalis (*p* = 0.011), non-iners *Lactobacillus* (*p* = 0.016), and *Mobiluncus curtisii* (*M. curtisii*) (*p* = 0.035) and the presence of *At. vaginae* (*p* = 0.049), *Bacterial Vaginosis-Associated Bacteria* (*BVAB*)-2 (*p* = 0.024), *Dialister microaerophilis* (*D. microaerophilis*) (*p* = 0.011), and *Prevotella amnii* (*P. amnii*) (*p* = 0.044) were associated with spontaneous PTB [36]. Moreover, in a prospective study, a higher frequency of CST-I assignment was observed in the term group compared to the preterm both at 20–22 weeks gestation and 26–28 weeks gestation (40.32 vs. 16.66% *p* = 0.0002 and 20.69 vs. 16.66% *p* = 0.03, respectively). Also, a diminished proportion of CST-V dominant microbiota was present in the term compared to the preterm group at both time points (9.68 and 22.22% *p* = 0.0002 and 10.34 and 25% *p* = 0.03, respectively). In addition, at 26–28 weeks gestation CST-II was assigned to 28% of term patients and to no patients in the preterm group (*p* < 0.0001) [68].

Assigning vaginal microbiomes in CSTs constitutes the basis for a more systematic analysis, but taxonomic composition analysis can provide further information. For example, a retrospective study concluded that the absence of elevated numbers of Operational Taxonomic Units (OTUs) of *L. iners* and *L. jensenii* might be the main difference between women delivering at term compared to PTB and might serve as a biomarker for PTB prediction [35]. Accordingly, to the aforementioned results, a cross-sectional study indicated that in vaginal specimens obtained at 16 weeks gestation, the dominance of *L. iners* was significantly higher in early PTB cases compared to term pregnancies (*p* = 0.003), while *L. Crispatus*-dominant microbiomes were more frequently derived from the control group (*p* = 0.009) [45]. The association of *L. iners* with PTB was also identified by a prospective study (*p* < 0.001), which also added that the combination of two or more *Lactobacillus* species had a beneficial impact on pregnancy duration [53]. The relative abundance of various *Lactobacillus* species was investigated by a prospective study that used a PCR array specific for 15 bacteria, and no significant association was observed with PTB, whereas high numbers of *L. crispatus*, *L gasseri*, *or L jensenii* were negatively associated with sPTB (*p* = 0.05), while at the same time, the detection of *L. gasseri* was predictive of term birth (*p* = 0.017) [52].

Besides *Lactobacillus* spp. relative abundance evaluation, microbiome analysis aims, also, at detecting differences in other microbial taxa. For instance, diversity in microbial composition was higher in the PTB group, and the relative abundances of several taxa including BVAB1, *Prevotella* cluster 2 (PC-2), and *S. amnii* were also higher in the PTG arm, reaching statistical significance (*p* < 0.05). In swabs collected early in pregnancy (6–24 weeks gestation), the presence of *Megasphera type 1* and TMP-H1 were associated with PTB. In fact, the incorporation of *S. amnii*, *PC*-2, TMP-H1, and BVAB1 in an early prediction model for PTB in swabs collected at 24 weeks gestation or earlier exhibited a sensitivity of 77.4% and specificity of 76.3% [38].

From the analysis of the longitudinal samples from a prospective study, it was concluded that women in the PTB group experience an increase in *Prevotella buccalis* (*p* < 0.0001), while women delivering at term have a significant increase in *L. crispatus* and *Finegolidia* (*p* = 0.0131 and *p* < 0.0001, respectively) [46]. Several other taxa, including *Gardnerella* spp., *M. curtsii/mulieris*, and *Sneathia sanguinegens*, with *M. curtsii/mulieris*, genus *Atopobium*, and genus *Megasphaera* exerting a significant association with PTB [47,65,74]. Interestingly, a case–control study expanding the analysis beyond the bacterial to the viral composition of the vaginal environment concluded that among *Papillomaviridae*, *Polyomaviridae*, *Herpesviridae*, *Poxviridae*, *Adenoviridae*, and *Anelloviridae*, no virus or viral group was found to be associated with PTB. However, increased viral richness was a predictor of PTB risk (*p* = 0.0005), and conversely, low viral richness was correlated with term birth (*p* = 0.03). Also, having both bacterial and viral diversity during the first trimester was a significant predictor of PTB (RR 3.12 95% CI 1.00–9.83 *p* = 0.04) [62].

#### 3.1.2. Miscarriage

Miscarriage is a common issue in obstetrics, complicating about 25% of pregnancies worldwide and leading to an estimated average of 44 pregnancy losses per minute [75]. Miscarriages can be divided into two categories based on the time point of occurrence: early miscarriages, happening before 12 weeks gestation, and late miscarriages, happening between 12 and 22 weeks gestation. Also, recurrent miscarriage, defined as three or more consecutive miscarriages, constitutes another variation of the condition [76,77].

Many studies attempted to elucidate the association between vaginal microbial composition and miscarriage. First, women experiencing miscarriage seem to have a vaginal microbiome characterized by increased diversity (*p* = 2.33 × 10^−8^) and richness (*p* = 0.0005) compared to women with healthy ongoing pregnancies. This diversity might derive from differences in abundance rates of *Bacteroides plebeius*, *B. breve*, *G. vaginalis*, and *M. girerdii* and *L. iners*, *Gardnerella*, *and Prevotella*, respectively, but no analysis for statistical significance regarding taxonomy was conducted in these studies [40,56]. Moreover, a relatively large prospective study observed that miscarriage in both the first and second trimester is associated with *Lactobacillus* species depletion (*p* = 0.0053), while the predominance of CST-IV was significantly different in the miscarriage group compared to the control group (*p* = 0.031). Interestingly, the *Lactobacillus* species depletion and the high bacterial diversity precede the diagnosis of miscarriage [28].

Furthermore, based on the results of the alpha and beta diversity analysis, which revealed an increased microbial diversity in miscarriage cases (Shannon index 5.48 vs. 5.18 *p* = 0.02), a prospective study attempted to discover the origins of this intergroup diversity. Although *Lactobacillus* was the predominant species in both arms, the relative abundance was lower in the case than in the reference group (16.51% vs. 23.00% *p* < 0.05), and simultaneously, a depleted abundance of *L. jensenii* and *L. gasseri* was observed in the miscarriage group (*p* = 0.00078 and *p* = 0.00069, respectively). Additionally, differences in the microbial diversity were attributed to greater abundance of *Mycoplasma genitalium* and *Ureaplasmas* (13.09% vs. 10.38% and 9.18% vs. 6.59%, respectively, both *p* < 0.05) in the reference group [59]. Increased *Ureaplasma* species rate, specifically *U. parvum*, has been, also, reported in another study, along with more frequent assignment to CST-III [33]. Contrarily, a prospective study concluded that elevated BVAB-3 log concentration in women experiencing miscarriage was the only significant dissimilarity between the two groups (4.27 vs. 3.71 *p* = 0.012). More specifically, in women aged <21 years, one unit increase in the BVAB-3 log concentration wound elevated the risk of miscarriage by 67.8% [78]. The same authors conducted the largest prospective relative study and concluded that the outcome of interest, meaning the second-trimester pregnancy loss, was significantly associated with diminished loads of *Lactobacilli* early in pregnancy, even after adjusting for confounding factors (HR 1.32 95% CI 1.10–1.64) [66].

Recurrent miscarriage (RM) has been investigated by several studies as a different outcome in non-pregnant women. The first study to ever inquire into this question employed the alpha diversity assessed by richness (ACE), Simpson, and Chao diversity indices to assess alpha diversity and no statistically significant differences occurred. Only bacterial richness was higher in the RM group, meaning that more OTUs were detected in the case group. After taxonomic composition analysis, it was indicated that *Atopobium*, *Prevotella*, and *Streptococcus* taxa were significantly more abundant in the miscarriage group, whereas *Lactobacillus* and *Gardnerella* were more commonly found in the control group [69]. In agreement with these results comes a case–control study that revealed that *Lactobacillus iners* was significantly decreased while *Ruminococcaceae*_UCG-005 and *Anaerococcus hydrogenalis* were significantly more abundant in the RM group (*p* < 0.05). Two taxa had significantly higher relative abundances in the control group, including *Lactobacillus* and *Gardnerella* [44]. In partial contrast with the two studies above comes a cross-sectional study reporting decreased rates of *Lactobacillus* species in the RM group but increased relative abundance of *Garnerella vaginalis*, *Prevotella bivia*, and *Porphyromonas* spp. (*p* < 0.05) [31]. Elevated *G. vaginalis* rates have been again reported in the RM arm compared to a healthy control arm of a cross-sectional study (8.7% vs. 5.7% *p* = 0.001) [54]. Using women with medically induced abortion as controls, another cross-sectional study showed that alpha diversity is increased in RM women (*p* < 0.05), and at the genus level, the expressive abundance of *Pseudomonas*, *Roseburia*, *Collinsella aerofaciens*, and *Arthrobacter* is higher [37]. Only one study concluded that although there was a significant difference in beta diversity (*p* = 0.036) neither the alpha diversity nor the taxonomic composition analysis of the vaginal microbiome revealed significant dissimilarities between the groups [49].

#### 3.1.3. Gestational Diabetes Mellitus (GDM)

Gestational diabetes Mellitus (GDM) constitutes a major burden not only on the pregnant woman’s well-being but also on the health care system, with a prevalence of approximately 5–20% [79]. The pathophysiology of GDM is partially known, with alterations in the hormonal and metabolic profile during pregnancy leading to a decrease in insulin sensitivity, which can sometimes result in the development of glucose intolerance and GDM [80].

Given that the microbial composition of the vagina undergoes significant changes during pregnancy, some studies tried to identify a connection between GDM development and the vaginal microbiome. Cortez et al. reported that the vaginal specimens exhibited significant differences regarding alpha diversity, with the control group having lower richness and diversity indices (*p* < 0.01) and the GDM arm presenting a significantly higher abundance of the genera *Bacteroides*, *Veillonella*, *Klebsiella*, *Escherichia-Shigella*, *Enterococcus*, and *Enterobacter* (*p* < 0.01) [34]. With regard to fungal microbiome, increased richness, and evenness indices in the GDM group pointed out that women with GDM have a more diverse fungal flora than healthy controls. This diversity was derived from the fact that in GDM-positive women, except for *Candida* and *Saccharomyces*, which were the most predominant fungal species, the fungal load attributed to uncultured fungi was significantly lower than in the control arm (*p* < 0.01) [70]. However, one study revealed no variations in the vaginal microbiome between women with GDM and healthy comparators [81].

#### 3.1.4. Preeclampsia

Preeclampsia is a major complication of pregnancy associated with fetal prematurity and long-term maternal cardiovascular morbidity [81]. It is estimated that it affects approximately 4.6% of pregnancies globally [82].

Only one study assessed the effect of vaginal microbiome aberrations on severe preeclampsia (SPE) development. In the context of alpha diversity, the two groups had similar richness but since Shannon and Gini-Simpson indices were higher in cases with SPE (*p* = 0.001 and *p* < 0.001, respectively), it was concluded that they had greater diversity than the controls. At the phylum level, the relative abundance of *Bacteroides* was significantly elevated in the SPE group (3.13% vs. 0.18% *p* = 0.015). After multivariable logistic regression at the genus and species level, the *Prevotella* genus and more specifically *P. bivia* were significantly associated with SPE [48].

#### 3.1.5. Chorioamnionitis (CAT)

Chorioamnionitis can present in 4% of full-term births, but histological CAT can be detected in up to 94% of deliveries occurring between 21 and 24 weeks gestation [83]. Since CAT is caused by ascending infection originating from the cervical and vaginal area, alterations in the vaginal microbiome can possibly be associated with CAT development [67].

Only one study investigated the association between CAT and vaginal microbiome composition. In this prospective observational study, the alpha diversity analysis indicated that the clinical chorioamnionitis group was characterized by a richer and more diverse vaginal microbiome compared to the healthy control group. Also, the relative abundance of *L. crispatus* was significantly higher in the control group, and conversely, after logistic regression analysis, it was found that the depletion of *L. crispatus* was associated with a higher risk of clinical CAT [42].

#### 3.1.6. Ectopic Pregnancy

Ectopic pregnancy rupture accounts for 5–10% of all pregnancy-related deaths, and tubal pregnancy, meaning a gestational sac that implants in the salpinx, constitutes the most common type of ectopic pregnancy [84]. Women with confirmed ectopic pregnancy exhibited a greater alpha diversity, as indicated by the Shannon diversity index (1.43 vs. 0.99 *p* = 0.03). Additionally, setting a relative abundance threshold of 85% for *Lactobacillus*, the researchers classified the vaginal microbiomes as *Lactobacillus*-dominated and *Non-Lactobacillus-Dominated Vaginal Microbiota* (NLDM). After adjusting for confounding factors, they concluded that there is a positive association between NLDM and ectopic pregnancy (OR 4.42 95% CI 1.33–14.71 *p* = 0.02) [55].

#### 3.1.7. Preterm Premature Rapture of Membranes (PPROM)

The prevalence of PPROM varies globally from 1–4% and its association with PTB has been well established, contributing to virtually 30–40% of premature births [85,86]. One study attempted to provide insight into the relationship between the characteristics of the vaginal microbiome and PPROM. The bacterial composition in the context of alpha diversity analysis differed significantly in richness, evenness, and diversity between the two groups. Also, increased relative abundance of *L. iners*, *G. vaginalis*, *P. bivia*, *Ochrobactrum* sp, *Prevotella timonensis*, and *Ureaplasma parvum* and decreased relative abundance of *L. gasseri* were correlated with PPROM [86].

## 4. Discussion

The vaginal microbiome constitutes a complex ecosystem constructed by epithelial cells, immune system cells and mediators, and microorganisms interacting in complex ways [87]. Given that the disruption of *Lactobacillus* dominance and high diversity can facilitate the colonization by pathogenic microbial taxa, for a pregnancy to progress uncomplicated to full term, it is of high importance that the microbiome retains its composition [88]. The specific mechanism linking alterations in the vaginal microbiome with different pregnancy complications is yet unclear but innovative laboratory techniques and study designs have offered some relevant indications. First, the depletion of beneficial bacteria loads, such as *Lactobacilli*, and an increase in pathogens result in the formation of a dysbiotic environment, which leads to the inflammation of the cervix and surrounding tissues. This inflammation can undermine the integrity of cervical tissues and contribute to cervical insufficiency development [63]. In addition, changes in the bacterial composition have been associated with conditions such as bacterial vaginosis (BV), which might be linked to miscarriage [89], and Vulvovaginal candidiasis (VVC), which is a type of infection that occurs in the mucous membranes and is caused by opportunistic microorganisms combined with various physiological changes, including lower cellular immunity, raised hormone levels, reduced vaginal pH, and increased vaginal glycogen concentration [90], Candida colonization is believed to be a result of using broad-spectrum antibiotics and has been linked to a reduced presence of *Lactobacillus*, perhaps due to the disruption of epithelial binding sites [91]. When there is a disruption in the equilibrium between *Candida*, the normal bacterial flora, and the immune defense mechanisms, colonization is replaced by infection [90,92,93].

As regards GDM, the dysbiotic vaginal environment and the consequent release of pro-inflammatory cytokines are hypothesized to increase insulin resistance and impair glucose tolerance, contributing to the development of GDM [94]. Moreover, in the context of hypertensive disorders of pregnancy, especially preeclampsia, the increased release of TNF-a in the maternal bloodstream induced by the inflammatory response to the altered microbiota might be associated with ischemic placental injury and arterial stiffness, leading eventually to the pathogenesis of this condition [95,96].

Last but not least, the link between the decreased rates of Lactobacillus species and the increased relative abundance of *Gardnerella*, *Prevotella*, *Atopobium*, *Sneathia*, and *Megasphaera* with ectopic pregnancy can be explained by two proposed mechanisms. First, the depletion of *Lactobacillus* populations increases the risk of urinary tract infections (UTIs). UTIs primarily affect the bladder, causing cystitis, and the urethra, causing urethritis [97]. UTIs constitute a major risk factor for ectopic pregnancy due to fallopian tube scarring [98,99]. Second, some of the aforementioned non-*Lactobacillus* species produce sialidase, a virulence factor that makes pregnant women vulnerable to ascending infections and consequent inflammation and fibrosis of the reproductive tract [100,101].

With regard to PTB, it has been clear that increased diversity of the vaginal microbiome and reduced relative abundance of *Lactobacillus* species are associated with high risk of PTB [30,32,51]. Also, the increased prevalence of *Gardnerella*, *Prevotella*, *Megasphera*, *Sneathia*, *Atopobium*, and *BVABs* seem to be associated with a shorter duration of pregnancy, while *L. crispatus* might have a protective role regarding pregnancy. Since the first are components of CST-IV, while the latter is the dominant species in CST-I, it is not surprising that CST-IV and CST-I are associated with preterm and term birth, respectively [36,45]. Since *Prevotella* and *Gardnerella* trigger the immune response by promoting proinflammatory cytokine production and are correlated with PPROM, it can be expected that they can affect the risk of PTB [86,102]. Lowered *Lactobacillus* loads and increased alpha diversity and relative abundance of non-*lactobacilli* bacteria are not only observed in PTB but also in miscarriage, GDM, ectopic pregnancy, CAT, and preeclampsia. This is attributed to virulence factors produced by these non-*lactobacilli* bacteria, such as sialidase, hyaluronidase, or IgA protease, which activate inflammatory pathways that probably constitute the starting point of these complications [103,104].

Despite the fact that the mechanism through which the vaginal microbiome exerts its impact on pregnancy outcomes is not totally understood, since, among the factors that can modify it, antibiotics and pre- and probiotics are included, an intervention strategy could be designed [105]. It has been already proven that probiotic *lactobacilli* reduce BV recurrence and can increase *lactobacilli* abundance [106]. However, the use of *Lactobacillus* and *Bifidobacterium* as probiotics in pregnant women seemed to have no effect on gestational age [107]. Moreover, the reliable establishment of certain taxa or CSTs associated with pregnancy complications can contribute to the formation of a microbial signature in high-risk pregnancies and could possibly be utilized to promptly intervene [108].

Hence, the disturbance of *Lactobacillus* dominance and the presence of a wide range of microbial species can promote the establishment of pathogenic bacteria that could possibly develop antimicrobial resistance (AMR), making the design of innovative and effective antimicrobials such as metals [109] and bacteriocins [110] as well as other approaches including antivirulence strategies [111] and phage therapy [112] urgently needed for humanity to combat life-threatening infections that are resistant to existing treatments [113].

This study has several limitations that merit careful consideration. First, relatively small sample sizes included in several of the studies jeopardize the reliability and the external validity of the results. In addition, most of the evidence included comprised cross-sectional and retrospective cohort studies, two study types both associated with selection bias. Furthermore, there were several factors regarding microbial analysis in the included studies that could have influenced the results, such as sample collection, extraction methods, and hypervariable regions used for sequencing. Among the included studies, there was great heterogeneity regarding the vaginal sampling, with some studies not even defining the sampling time point. Finally, the racioethnic composition of the cohorts is a factor that could have altered the effect size of vaginal microbiome alterations on pregnancy complication incidence.

## 5. Conclusions

To summarize, pregnancy complications pose a major burden on maternal and fetal health and well-being. New approaches are being applied in order to not only trace women at high risk of developing these disorders but also to develop appropriate prevention strategies. One of these approaches is facilitated through the analysis and the potential of the maternal reproductive tract microbiome. Given that high diversity and richness, a depleted predominance of *Lactobacillus* species, and an increased abundance of specific taxa, such as *Gardnerella* and *Prevotella*, have been associated with several pregnancy complications indicates that the vaginal microbiome constitutes a possible target. However, it is important that more high-quality studies with greater racioethnic diversity and more homogenous designs with regard to sampling time points and sequencing techniques are conducted.

## Figures and Tables

**Table 1:** Summary of the included studies. MC, miscarriage; PTB, preterm birth; RMC, recurrent miscarriage; GDM, gestational diabetes mellitus; CAT, chorioamnionitis; PE, preeclampsia; EP, ectopic pregnancy; PPROM, preterm premature rupture of membranes.

Study	Study Design	Country	Time Period	Complication	Summary of Results
[28]	Prospective observational	UK	Mar 2014– Mar 2016	MC	Miscarriage is associated with *Lactobacillus* spp. depletion.
[29]	Retrospective observational	Peru	Oct 2013– May 2014	PTB	No association between CSTs and PTB.
[30]	Prospective observational	UK	Oct 2013– Jun 2015	PTB	Lactobacillus depletion and high diversity more commonly found in PTB arm.
[31]	Case–control study	Turkey	Jul 2019– Dec 2019	RMC	Low Lactobacillus and high Gardnerella and Prevotella prevalence in RMC patients.
[32]	Retrospective observational	US	-	PTB	Low Lactobacillus and high Gardnerella prevalence associated with PTB.
[33]	Prospective observational	Korea	Sept 2014– Aug 2018	PTB MC	PTB patients more commonly assigned to CST IV. Almost all MC patients were assigned to *L. iners*-dominated CST.
[34]	Cross-sectional	Brazil	Jan 2014– Jan 2016	GDM	GDM presented a significantly higher abundance of the genera Bacteroides, Veillonella, Klebsiella, Escherichia-Shigella, Enterococcus, and Enterobacter.
[35]	Retrospective observational	Brazil	May 2014– Mar 2016	PTB	Elevated number of *L. iners* and *L. jensenii* OTUs in PTB.
[36]	Cross-sectional	US	-	PTB	CST III and CST IV were associated with PTB.
[37]	Cross-sectional	China	Jan 2010– Dec 2016	RMC	High alpha diversity and elevated abundance of specific genera, such as Pseudomonas, in women experiencing RMC.
[38]	Cross-sectional	US	-	PTB	High levels of *S. amnii*, Prevotella, TMP-H1, and BVAB1 can predict PTB.
[39]	Retrospective observational	Canada	-	PTB	Women delivering at term were less likely to be positive for Mycoplasma and Ureaplasma.
[40]	Cross-sectional	Russia	-	MC	The abundance of *B. plebeius*, *B. breve*, *G. vaginalis*, and *M. girerdii* was significantly higher in the miscarriage group.
[41]	Case–control study	Kenya	Mar 2018– Mar 2019	PTB	There were no statistically significant differences in either the taxa or the CST assignment.
[42]	Prospective observational	China	-	CAT	The clinical CAT group was characterized by a richer and more diverse vaginal microbiome compared to the healthy control group.
[43]	Prospective observational	US	-	PTB	There were no statistically significant differences in either the taxa or the CST assignment.
[44]	Case–control study	China	-	RMC	*L. iners* was significantly decreased while Ruminococcaceae and Anaerococcus were significantly more abundant in RMC cases.
[45]	Cross-sectional	UK	Jan 2013– Aug 2014	PTB	*L. iners* was significantly higher in early PTB cases compared to term pregnancies.
[46]	Prospective observational	Qatar Thailand	-	PTB	PTB was significantly associated with CST-IVB presenting even from the first trimester of the pregnancy. Women in the PTB group experienced an increase in P. buccalis.
[47]	Retrospective observational	US	-	PTB	Megasphaera, *Gardnerella* spp., and Atopobium vaginae are associated with PTB.
[48]	Case–control study	Taiwan	-	PE	Prevotella genus and more specifically *P. bivia* were significantly associated with severe PE.
[49]	Case–control study	China	-	MC	Increased diversity and CST IV were associated with miscarriage.
[49]	Case–control study	China	-	RMC	There were no statistically significant differences in either the taxa or the CST assignment.
[50]	Retrospective observational	China	-	PTB	There were no statistically significant differences in either the taxa or the CST assignment.
[51]	Prospective observational	Nigeria	Dec 2018– Sep 2019	PTB	Increased abundance of Atopobium, Gardnerella, and Prevotella and CST IV assignments were observed in PTB group.
[52]	Prospective observational	Australia	Jul 2015– Dec 2017	PTB	High numbers of *L. crispatus*, *L gasseri*, or *L jensenii* were negatively associated with sPTB.
[53]	Prospective observational	Austria	-	PTB	The dominance of *L. iners* was significantly different in women delivering preterm.
[54]	Case–control study	Finland	Mar 2018– Jun 2020	RMC	The relative abundance of *G. vaginalis* was significantly higher in the RMC group.
[55]	Case–control study	China	May 2018– Dec 2018	EP	There is a positive association between NLDM and ectopic pregnancy (*p* = 0.02).
[56]	Prospective observational	Australia	Oct 2018– Apr 2019	MC	Vaginal microbiome deriving from patients with miscarriage was elevated.
[57]	Prospective observational	US	Jul 2008– Sep 2011	PTB	Women delivering at term were mostly assigned to CST IV, while women from the PTB arm were mostly assigned to CST III.
[58]	Case–control study	US	2012–2015	PTB	There were no statistically significant differences in either the taxa or the CST assignment.
[59]	Prospective observational	China	Nov 2018– Nov 2019	MC	Decreased *L. jensenii* and *L. gasseri* and increased *M. genitalium* and Ureaplasma rates were observed in the MC group.
[60]	Case–control study	Canada	-	PTB	Lactobacillus species dominance might be associated with low risk of early but not late PTB.
[61]	Case–control study	China	-	GDM	No variations of the vaginal microbiome were observed between the two groups.
[62]	Case–control study	US	2012–2015	PTB	Having both bacterial and viral diversity during the first trimester was a significant predictor of PTB.
[63]	Cross-sectional	China	Jan 2019– Apr 2020	PPROM	*L. iners*, *G. vaginalis*, *P. bivia*, *P. timonensis*, *U. parvum*, and *Ochrobactrum* spp. were associated with PPROM.
[64]	Prospective observational	France	Jul 2007– Apr 2012	PTB	*A. vaginae* levels of equal or more than 10⁸/mL were correlated with PTB before 22 weeks of gestation.
[65]	Case–control study	US	-	PTB	In the first trimester, increased rates of *M. curtsii*/*mulieris* increased the risk of PTB.
[66]	Prospective observational	US	Sep 2001– Jun 2004	MC	Second-trimester pregnancy loss was significantly associated with diminished loads of Lactobacilli early in pregnancy.
[57]	Prospective observational	US	Jul 2008– Sep 2011	MC	Elevated BVAB3 log concentration in women experiencing miscarriage was the only significant dissimilarity between the two groups.
[67]	Case–control study	US	-	PTB	There were no statistically significant differences in either the taxa or the CST assignment.
[68]	Prospective observational	UK	-	PTB	Term and pre-term groups were assigned more frequently to CST I and CST V, respectively.
[69]	Case–control study	China	Sep 2016– Mar 2017	RMC	Atopobium, Prevotella, and Streptococcus taxa were significantly more abundant in the miscarriage group.
[70]	Prospective observational	China	Feb 2012– Jun 2012	GDM	Women with GDM have a more diverse fungal flora than healthy controls.

## Data Availability

No new data were created or analyzed in this study. Data sharing is not applicable to this article.
